# Circulating immune cells in patients with surgically resected nonfunctional pancreatic neuroendocrine tumors

**DOI:** 10.1186/2051-1426-3-S2-P140

**Published:** 2015-11-04

**Authors:** Zeljka Jutric, Jan Grendar, Benjamin Cottam, Chet Hammill, Ronald Wolf, Paul Hansen, Marka Crittenden, Michael Gough, Pippa Newell

**Affiliations:** 1 Providence Cancer Center, Portland, OR, USA; 2 Earle A. Chiles Research Institute, Portland, OR, USA; 3 The Oregon Clinic, Portland, OR, USA

## Background

There is debate regarding whether surgical resection and/or lymphadenectomy are indicated for small nonfunctional pancreatic neuroendocrine tumors (PNETs). Myeloid cell population expansion in peripheral blood has been correlated with clinical stage of patients with solid tumors. We aim to determine if blood sampling can be used as a predictor of malignant potential in nonfunctional PNETs.

## Methods

We prospectively measured cell counts in 29 patients with PNET using flow cytometry of fresh whole blood before and after surgery, including CD3+, CD8+, and CD4+ T cells; monocytes; and granulocytes. Correlations were sought with known clinical markers of malignant potential such as tumor size, grade, number of positive lymph nodes, and TNM stage using t-test and one-way ANOVA analysis (STATA Inc).

## Results

In this small cohort of patients, there were no differences in circulating immune cell counts between patients with lymph node metastases versus those without, nor in patients with tumors > 2cm, nor in those patients with high grade tumors. Interestingly, patients with lymph node metastases had a significant decrease in number of circulating CD8+ T cells post operatively when compared to those with negative lymph nodes (p = 0.013). There was a similar decrease in monocyte count post operatively in patients with positive versus negative lymph nodes that did not reach significance (p = 0.056).

## Conclusions

Our data do not provide evidence that circulating immune cell populations can be used as biomarkers of lymph node positivity or of malignant potential in PNET prior to resection.

